# A Non-Linear Model of an All-Elastomer, in-Plane, Capacitive, Tactile Sensor Under the Application of Normal Forces

**DOI:** 10.3390/s18113614

**Published:** 2018-10-24

**Authors:** Kourosh M. Kalayeh, Panos G. Charalambides

**Affiliations:** Department of Mechanical Engineering, The University of Maryland, Baltimore County, Baltimore, MD 21250, USA; kourosh2@umbc.edu

**Keywords:** analytical modeling, capacitive all-elastomer tactile sensors, finite flat punch indentation, finite elements, inverse analysis

## Abstract

In this work, a large deformation, non-linear semi-analytical model for an all-elastomer, capacitive tactile unit-sensor is developed. The model is capable of predicting the response of such sensors over their entire sensing range under the application of normal forces. In doing so the finite flat punch indentation model developed earlier is integrated with a capacitance model to predict the change-in-capacitance as a function of applied normal forces. The empirical change-in-capacitance expression, based on the parallel plate capacitance model, is developed to account for the fringe field and saturation effects. The elastomeric layer used as a substrate in these sensors is modeled as an incompressible, non-linear, hyperelastic material. More specifically, the two term Mooney-Rivlin strain energy function is used as a constitutive response to relate the stresses and strains. The developed model assumes both geometrical as well as material non-linearity. Based on the related experimental work presented elsewhere, the inverse analysis, combining finite element (FE) modeling and non-linear optimization, is used to obtain the Mooney-Rivlin material parameters. Finally, to validate the model developed herein the model predictions are compared to the experimental results obtained elsewhere for four different tactile sensors. Great agreements are found to exist between the two which shows the model capabilities in capturing the response of these sensors. The model and methodologies developed in this work, may also help advancing bio-material studies in the determination of biological tissue properties.

## 1. Introduction

Owing to advances in robotics and bionic technologies in the past few decades, tactile sensors have been the subject of intense attention [1,2,3]. Tactile sensors, through texture identification and tactile perception, are capable of providing haptic feedback which is essential in object characterization and dexterous manipulation. Therefore, in recent years, in addition to tactile sensor design and development, a lot of attention has been given to research in texture identification. In such works, the focus is on tactile information processing and tactile learning instead of the technological aspects of these devices. For example, Watanabe et al. [4] developed a multi-axial tactile sensor using micro-cantilevers embedded in a soft elastomeric layer. They showed that their proposed design is capable of identifying three various kind of papers. In another example, Xu et al. [5] developed an algorithm based on Bayesian exploration, and integrated it with multimodal tactile sensors (BioTac from SynTouch, Los Angeles, CA, USA) to make an exploratory movements similar to those of humans in order to identify objects. In another work by Friedl et al. [6], the authors, inspired by the biology of human tactile perception, implemented a neurorobotic texture classifier to classify surface textures by touch. More recently, Kaboli et al. [7] proposed a set of novel tactile descriptors to extract robust features from generated raw tactile signals. They then evaluated their proposed tactile descriptors using their target search object algorithm developed in [8].

Robotic smart skins using these tactile sensors are, therefore, expected to increase dexterity and perceptual capabilities of robots. This, in turn, will result in more precise and safer interaction with humans and other objects in an unstructured and complex environment [9,10]. The development of sophisticated smart skin is also expected to have transformative impacts on bionics.

Recent advances in microelectromechanical system (MEMS) technologies enable batch fabrication and development of micron-size sensors. These unit sensors can then be arranged in an array, like pixels in an image, to provide high spatial resolution. In the past, the common approach in fabricating these sensors was to use a metal, ceramic, or silicon diaphragm to detect local deformation [11,12,13]. The problem with using metal and silicon as a substrate is they lack flexibility [14]. For these sensors to be able to be mounted on curved surfaces, they need to be highly stretchable and flexible [15]. Thus, flexible substrates of various polymer-based materials, such as parylene, polyimide (PI), or polydimethlysiloxane (PDMS) are proposed as the substrate for these sensors [16,17,18,19].

Different sensing mechanisms such as resistive [20], piezoresistive [21,22], capacitive [23], and piezoelectric [24,25] have been proposed for tactile sensors. Among these mechanisms, capacitive tactile sensors gain more attention because of their reliability, stability, simple structure, low-cost, and temperature independence [26,27,28].

While in recent years, there have been tremendous advances in designing and fabricating tactile sensors with both high dynamic range and high spatial resolution [29,30,31], little work has been done in modeling of such sensors. This is particularly due to an inherent non-linear behavior of the elastomeric materials used as a substrate in these sensors. The early works on modeling of tactile sensors are traced back to [32] where Phillips et al. developed a model of skin using the theory of continuum mechanics. They modeled the skin as a linear elastic and isotropic half-space. More general contact analysis of this kind can be found in [33]. In a similar work, Fearing and Hollerbach modeled the finger-object contact as an infinite linear elastic half-plane under the application of a point load [34]. Using the thin-plate theory, Maier-Schneider et al. [35] and Wang et al. [36] developed an analytical model for the load-deflection of square membranes, which can be used for tactile sensor model development. In more recent works, Liang et al. [37] developed an analytical model for a polymeric, capacitive, tactile sensors based on the Ritz method. In doing so, they simplified their six-layer sensor as a two-layer plate structure by assuming that the polyethylene terephthalate (PET) substrate of the sensor to be rigid. In [38], they modified their previous model by analyzing three-layer plate structure, accounting for the deformation of PET layers as well. In [39], based on [37,38], a new array of tactile sensors using the truncated pyramid as a dielectric layer was developed. The corresponding analytical model was also presented. Similar to [37,38], the Ritz method was used in the model development. In all of the above references the polymeric layer was modeled as a linear elastic isotropic material.

Another approach in modeling tactile sensors is using inverse analysis to reconstruct the contact force distribution based on the sensor output. For example in [40] Vásárhelyi et al. developed an analytical inverse solution for a tactile sensor under the application of a point load. In an another example Liu et al. [41] proposed an inverse solution for the contact force in a sparse tactile sensor array using the diffusion effect of the elastomer.

Due to the lack of robust and reliable analytical models, finite element analysis (FEA) is widely used to assess the performance of these sensors [42,43]. Even in FEA, because of the convergence issues associated with the non-linearity and large deformation of polymers, the elastomeric layer is often modeled as a linear elastic material limiting the model applications to small strain, small deformation conditions.

In light of the recent advances in machine learning (ML), there are some works trying to use ML algorithms like neural networks and support vector machines (SVM) to predict the response of tactile sensors under different loading conditions [44,45,46]. However, several problems are associated with the above approach which may include; the need for an empirical induction, the need for large amount of sample data, and the dependency of the quality of the model on the training data. In general, an analytical solution, whenever available, is preferred to ML solution [47].

As briefly described in the above, most available tactile sensor models treat the polymeric layer as a linear elastic and isotropic medium. Thus, they are limited to small deformation and small strain conditions. Inevitably, such assumptions limit the predictive capabilities of these models to the linear regime, and make them unreliable under large deformation conditions thus limiting their sensing range.

Novel all-elastomer tactile sensors have been designed and fabricated previously [48]. The purpose of the current study is to develop a large-deformation, large-strain model capable of predicting the response of the fabricated sensors under the application of normal forces covering their full sensing range. In doing so, a non-linear semi-analytical model developed for finite flat punch indentation of soft elastomeric layer in [49,50], will be integrated with an enhanced capacitance model. The empirical expression for change-in-capacitance is developed based on the parallel plate capacitance theory and through model comparisons to the experimental results. The enhanced capacitance model is expected to further improve model predictions and sensor design by accounting for fringe field and saturation effects.

## 2. Summary of the Finite Flat Punch Indentation Model

A novel all-elastomer, capacitive, tactile sensor was designed and fabricated as discussed in [48]. While the fabricated sensor in [48] is designed to sense both normal and shear forces, the focus in this study is to develop a model capable of predicting the response of the above sensor under the application of normal forces only. The shear sensor modeling remains the subject of an on-going research.

**Figure 1:**
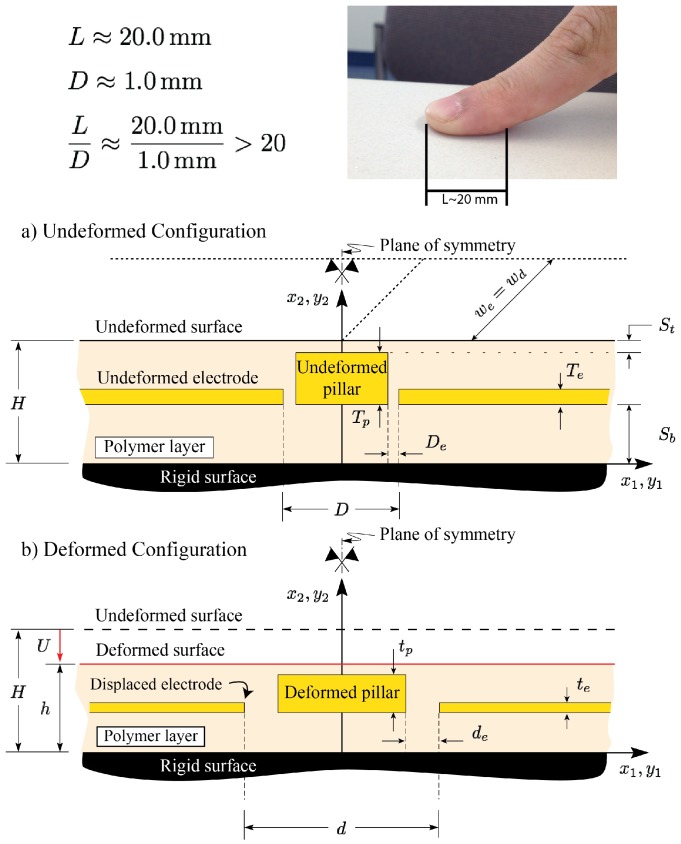
A schematic of the tactile unit-sensor designed and fabricated in [48]. Undeformed (reference) configuration is shown in (**a**); while the deformed (current) configuration is shown in (**b**). Due to typical large contact length-electrode spacing ratios, the mechanics of the elastomeric layer in the vicinity of the symmetry plane can be modeled as uniform compression of an infinitely long layer.

The working principle of the fabricated sensors in [48] is shown in Figure 1. As shown in the figure, the all-elastomer tactile unit-sensor consists of two electrodes and a pillar (conductive features) embedded in a polymeric layer (dielectric feature). As a force is applied on the sensor, its top-surface deforms and the sensor compresses in the direction of the applied force and expands in the transverse direction, consistent with Poisson’s effect. As a result, the distance as well as the overlapping area between the electrodes and pillar will change. This, in turn, will alter the stored electrical energy and the capacitance between the conductive features, consequently. Therefore, the sensor can manifest the applied loading as a change in capacitance between the electrodes and the change in capacitance can be used to identify the location, magnitude, and direction of the applied loads. An important premise of the above model is that the relatively stiffer conductive elastomer, during deformation, floats within the layer like a pair of stiff wires placed in a layer of soft jello [49].

**Figure 2:**
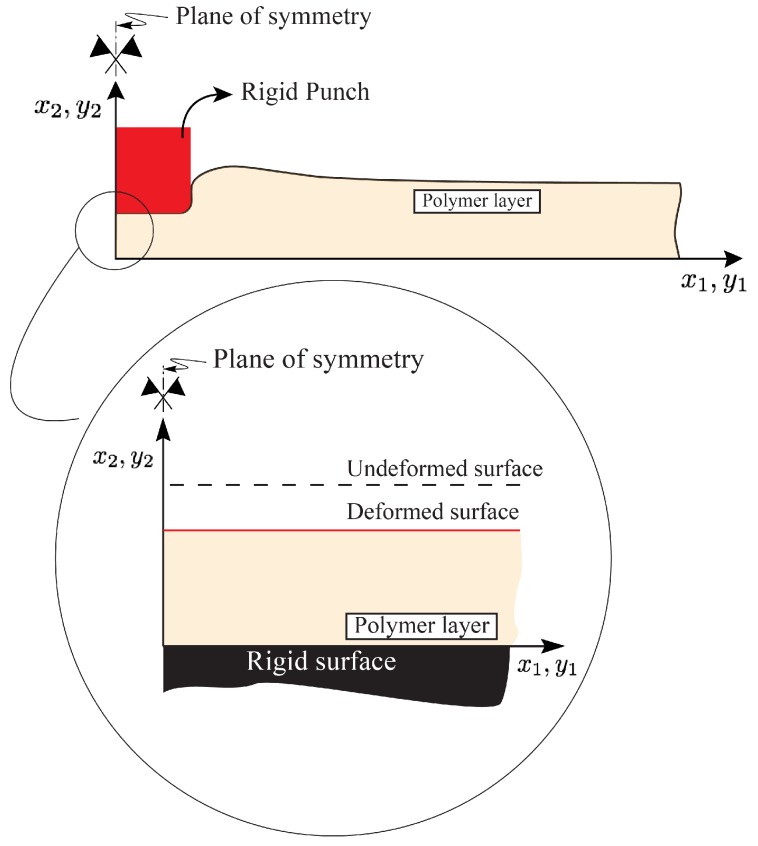
A schematic of an infinitely long elastomeric layer compressed by a finite flat punch. The schematic corresponding to the uniform compression can be seen as a close-up view of the indentation model in the vicinity of the symmetry plane.

As shown in the figure, a typical punch-sensor contact length is in the order of 20 mm while the largest distance between two electrodes in these sensors is in the order of 1.0 mm [48]. Therefore, as discussed in [49], the mechanics of the elastomeric layer in the vicinity of the symmetry plane i.e., the region of interest in the tactile sensor modeling, can be estimated by considering an infinitely long layer compressed uniformly (infinite layer-infinite punch assumption). The model for the above problem i.e., the uniform compression of a soft polymeric layer, has been solved analytically and was presented in [49]. Although the model developed in [49] was then adopted in the development of a capacitive tactile sensor predictive model [31], several approximations were introduced, thus limiting the effectiveness of the developed model. A major limiting aspect is that, for contact force calculations, a corresponding infinite layer-finite punch formulation (i.e., finite flat punch indentation problem) is needed. This problem, which is shown in Figure 2, was solved in [50] and shall be employed in this study in the development of an enhanced sensor model. Consistent with the figure, the schematic associated with the uniform compression case shown in Figure 1, can be seen as a close-up view of the schematic associated with the indentation case in the vicinity of the symmetry plane.

### 2.1. Layer Kinematics

**Figure 3:**
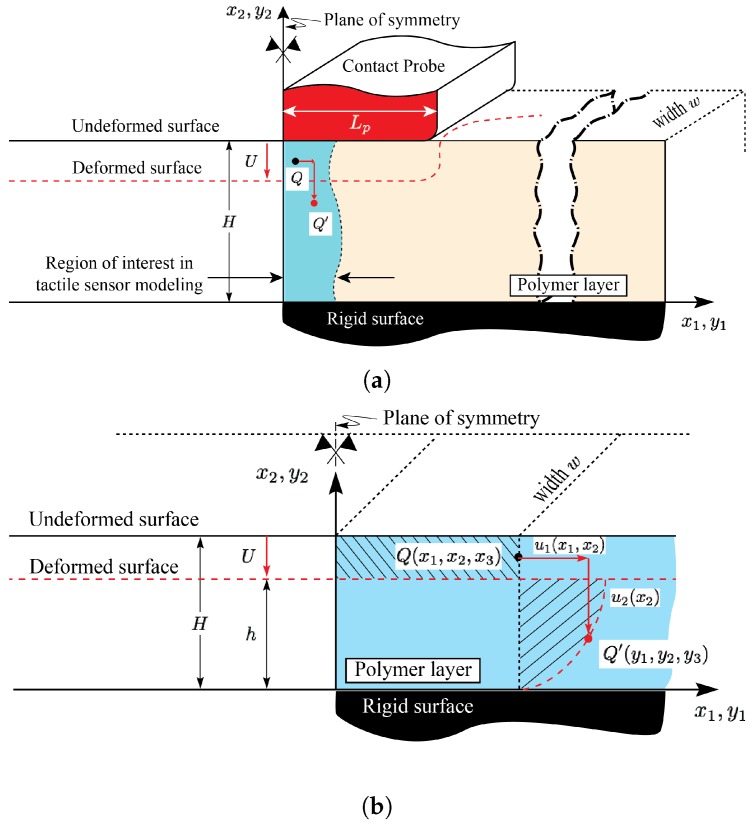
(**a**) A schematic of a boundary value problem associated with the finite flat indentation case. The region of interest in the tactile sensor modeling, in the vicinity of the symmetry plane, is highlighted; (**b**) Magnified view of the highlighted region in (**a**) which can be modeled as the uniform compression case. For clarity, the conductive features are not shown.

A schematic of a boundary value problem solved in [49,50] is shown in Figure 3. More specifically, a schematic of an infinitely long layer of finite thickness *H* compressed under the application of a finite flat punch of half-length $L_{p}$ is shown in Figure 3a. The region of interest in the tactile sensor modeling in the vicinity of the symmetry plane and far from the punch edges is highlighted. Consistent with the figure, the mechanics of the highlighted region, whose schematic is magnified in Figure 3b, can be modeled by considering a long layer of finite thickness *H* compressed uniformly under the applied displacement *U*. Provided the contact, loading, and material are uniform in the $x_{3}$ direction, the problem can be modeled under the plane strain condition. The two-term Mooney-Rivlin (M-R) constitutive response is used to model the elastomeric layer [49].

As shown in the figure, coordinates $\left(x_{1},x_{2},x_{3}\right)$ and $\left(y_{1},y_{2},y_{3}\right)$ are parametrizing the reference (undeformed) and current (deformed) configuration, respectively. Under the displacement components $u_{1}\left(x_{1},x_{2}\right)$ and $u_{2}\left(x_{2}\right)$, in the $x_{1}$- and $x_{2}$-directions, respectively, point $Q(x_{1},x_{2},x_{3})$ in the reference state deforms to point $Q^{\prime }\left(y_{1},y_{2},y_{3}\right)$ in the current state. Using the models developed in [49,50], the above displacement components are given by,

(1a) $$\begin{array}{c} u_{1}\left(x_{1},x_{2}\right)=y_{1}-x_{1}=x_{1}\left(f\left(x_{2}\right)-1\right), \end{array}$$

(1b) $$\begin{array}{c} u_{2}\left(x_{2}\right)=y_{2}-x_{2}=g\left(x_{2}\right)-x_{2}, \end{array}$$

where $f(x_{2})$ and $g(x_{2})$ are general functions of $x_{2}$ and are expressed as follows,

(2) $$f\left(x_{2}\right)=\cos\left(\alpha x_{2}\right)+\tan\left(\alpha H\right)\sin\left(\alpha x_{2}\right),$$

(3) $$\begin{array}{c} g\left(x_{2}\right)=\frac{\cos(\alpha H)}{\alpha }\left(\log\frac{1+\sin(\alpha H)}{1-\sin(\alpha H)}-\log\frac{1+\sin\left(\alpha H\right)-\cos\left(\alpha H\right)\tan\left(\frac{\alpha x_{2}}{2}\right)}{1-\sin\left(\alpha H\right)+\cos\left(\alpha H\right)\tan\left(\frac{\alpha x_{2}}{2}\right)}\right). \end{array}$$

In the above expressions, *H* is the initial layer thickness and α is the parameter that only depends on the layer deformation *U* and is given by the following empirical expression [50],

(4) $$\begin{array}{c} \alpha H=1.6718{\left(\frac{U}{H}\right)}^{0.5313}. \end{array}$$

### 2.2. Stress Analysis

Consistent with [49,50], the non-trivial Cauchy (true) stress components in the deformed (current) configuration developed in the polymeric layer are given by,

(5a) $$\begin{array}{c} \sigma_{11}=-P+C_{1}\left(f^{2}+x_{1}^{2}{f^{\prime }}^{2}\right)+C_{2}{g^{\prime }}^{2}, \end{array}$$

(5b) $$\begin{array}{c} \sigma_{12}=\mu_{0}x_{1}f^{\prime }g^{\prime }, \end{array}$$

(5c) $$\begin{array}{c} \sigma_{22}=-P+C_{1}{g^{\prime }}^{2}+C_{2}\left(f^{2}+x_{1}^{2}{f^{\prime }}^{2}\right), \end{array}$$

(5d) $$\begin{array}{c} \sigma_{33}=-P+C_{1}+C_{2}, \end{array}$$

where $C_{1}$ and $C_{2}$ are the M-R material constants, $\mu_{0}=C_{1}-C_{2}$ is the initial shear modulus, and $P(x_{1},x_{2})$ is the independent pressure term which is expressed as follows,

(6) $$\begin{array}{cc} P(x_{1},x_{2})= & -\frac{\mu_{0}}{2}\alpha^{2}x_{1}^{2}f^{2}+\frac{C_{1}+C_{2}}{2}\frac{1}{f^{2}}+C_{2}\left(x_{1}^{2}f^{\prime }{}^{2}+f^{2}\right)+\Pi . \end{array}$$

In the above equation, Π is a function of deformation level (*U*) and punch half length ($L_{p}$) and is given as follows [50],

(7) $$\begin{array}{c} \frac{\Pi }{\mu }\left(\frac{U}{H},\frac{L_{p}}{H}\right)=\left(\left(\zeta +\frac{L_{p}}{H}\right)\exp\left(\xi \frac{U}{H}\right)\right.\left.+\left(\zeta -\frac{L_{p}}{H}\right)\exp\left(-\xi \frac{U}{H}\right)\right)\frac{\Pi_{0}}{\mu }, \end{array}$$

with $\Pi_{0}=1.4559,\zeta =0.2056$ and ξ=2.6783, all obtained in [50].

Using the Cauchy stress, the non-trivial 1st Piola-Kirchhoff (P-K) stress components in the undeformed (reference) state can be expressed as follows [49],

(8a) $$\begin{array}{cc} T_{11} & =\frac{1}{f}\left(-P+C_{1}f^{2}+C_{2}\frac{1}{f^{2}}+C_{2}x_{1}^{2}{f^{\prime }}^{2}\right), \end{array}$$

(8b) $$\begin{array}{cc} T_{11} & =\mu_{0}x_{1}\frac{f^{\prime }}{f^{2}}x_{1}f^{\prime }\left(-P+C_{1}\frac{1}{f^{2}}+C_{2}\left(f^{2}+x_{1}^{2}{f^{\prime }}^{2}\right)\right), \end{array}$$

(8c) $$\begin{array}{cc} T_{22} & =\mu_{0}x_{1}f^{\prime }, \end{array}$$

(8d) $$\begin{array}{cc} T_{22} & =f\left(-P+C_{1}\frac{1}{f^{2}}+C_{2}\left(f^{2}+x_{1}^{2}f^{\prime }{}^{2}\right)\right). \end{array}$$

### 2.3. Contact Force Calculation

The contact force between the punch and the sensor in the $x_{2}$-direction can be calculated by either integrating the Piola-Kirchhoff stress component $T_{22}$ over the undeformed contact area or the Cauchy stress component $\sigma_{22}$ over the deformed contact area [49,50]. As discussed in [50], a special care needs to be taken in carrying out the above integration. More specifically, due to the slip of material points on the top surface of the polymeric layer, some of the points that were initially in contact with the horizontal surface of the punch slip and become in contact with the vertical edge of the punch. These points will not carry any $\sigma_{22}$ stress component, and therefore will not contribute to the reaction force developed in the punch in the $x_{2}$-direction. This needs to be accounted for in contact force calculations.

In light of the above and Consistent with [50], $L_{\mathrm{eff}}$ is defined as an effective half-length over which the $\sigma_{22}$ stress component is non-zero. Using the methodology described in [50], $L_{\mathrm{eff}}$ was found to take the following form,

(9) $$\begin{array}{c} L_{\mathrm{eff}}=\cos\left(\alpha H\right)L_{p}, \end{array}$$

where α is given by Equation (4) and depends on the deformation level, and $L_{p}$ is the punch half-length. By carrying out the integration the contact force in the $x_{2}$-direction can be found to be in the following form [49,50],

(10) $$\begin{array}{c} \begin{array}{c} F=2w_{p}L_{\mathrm{eff}}\lambda \mu_{0}\left(\frac{1}{6}\alpha^{2}L_{\mathrm{eff}}^{2}\lambda^{2}+\frac{1}{2}\frac{1}{\lambda^{2}}-\frac{\Pi }{\mu_{0}}\right), \end{array} \end{array}$$

where $w_{p}$ is the out-of-plane punch width, $L_{\mathrm{eff}}$ is the effective contact length and is given by Equation (9), λ=f(H) is the stretch ratio in the $x_{1}$-direction on the top surface of the polymeric layer, $\mu_{0}=C_{1}-C_{2}$ is the shear modulus, α is given by Equation (4), and Π is given by Equation (7) [50].

## 3. Tactile Unit-Sensor Model Development

**Figure 4:**
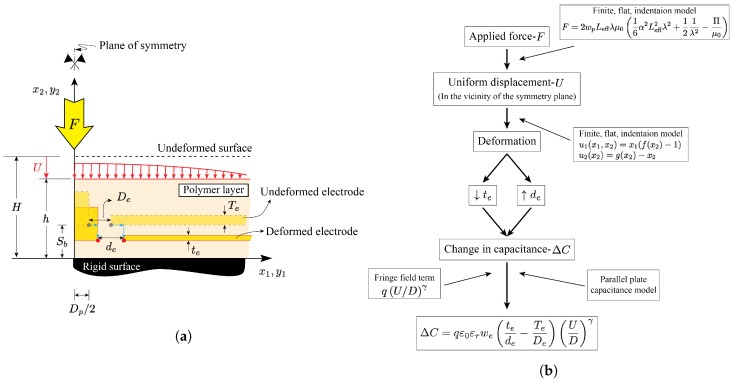
(**a**) A schematic of the half symmetric cross section of the tactile sensor designed and fabricated in [48] and modeled in this study; (**b**) A flowchart showing the process used in this study to model the relationship between the change-in-capacitance and applied force.

A schematic of the half symmetric cross section of the tactile sensor designed and fabricated in [48] is shown in Figure 4a. As shown in the figure, due to the applied force in the $x_{2}$-direction, the sensor in the vicinity of the symmetry plane undergoes a compressive stress. This, in turn, causes the sensor to compress in the $x_{2}$-direction and expand in the $x_{1}$-direction, consistent with the Poisson’s effect and mass conservation. A capacitance sensor model would require predictive capabilities related to the movement/deformation of the compliant electrodes as needed to estimate the capacitance between them.

As shown in Figure 4a, after the deformation, the initial electrode gap $D_{e}$ increases to a larger gap $d_{e}$, while the initial electrode thickness $T_{e}$ decreases to a smaller thickness $t_{e}$.

In the proceeding sections, the large deformation mechanics model developed in [49,50] is combined with an enhanced parallel plate capacitance model accounting for the fringe field as well as saturation effects to develop a tactile sensor model capable of predicting the applied force as a function of change in capacitance over the entire sensing range. The above process is summarized in the flowchart shown in Figure 4b.

### 3.1. Dimensions of Fabricated Sensors

**Figure 5:**
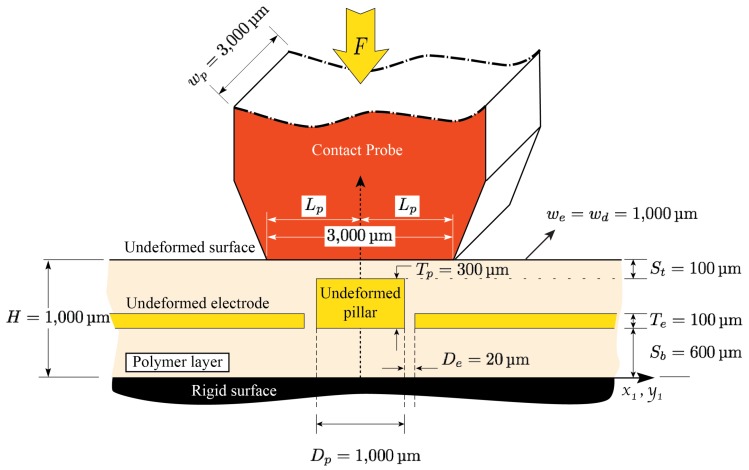
A detailed geometry of the tactile unit-sensor designed and fabricated in [48].

A schematic showing the detailed geometry of the fabricated sensor in [48] is shown in Figure 5. As shown, through a special microfabrication process, conductive layers (electrodes and pillar) are embedded into the polymeric layer of initial thickness *H* = 1000 μm. The conductive and dielectric layers have out-of-plane width $w_{e}=w_{d}$ = 1000 μm. The electrodes and pillar are of initial thickness $T_{e}=100\;\mu m$ and $T_{p}=300\;\mu m$, respectively, and are placed at $S_{b}=600\;\mu m$ from the bottom surface of the sensor. The initial electrode gap of $D_{e}=20\;\mu m$ is introduced between the electrodes and pillar. The pillar has a total in-plane width of $D_{p}$ = 1000 μm making the total distance between the electrode and the symmetry plane to be 52670μm. Experimental tests are performed by applying a vertical displacement to the sensor using a 3×3mm probe and reading the capacitance as well as the reaction forces [48].

### 3.2. Experimental Results

It is important to note that this study is not an experimental study. It is mainly focused on the development of an enhanced model capable of predicting tactile sensor response over its entire sensing range under large deformation conditions. However, in calibrating the model existing experimental results are employed. Thus, the experimental results reported in [48] will be used for (a) performing an inverse analysis as needed to obtain the M-R parameters; (b) obtaining the capacitance model parameters; and (c) validating the modeling results. Therefore, for completeness, the related experimental results reported in [48] are summarized in this section. More specifically, the results associated with the response of the sensors under the application of normal force are presented in Table 1. As shown in the table, the change in capacitance (ΔC) and the applied contact force (*F*) are tabulated for each top-surface deformation level (*U*) tested in [48].

### 3.3. Tactile Unit-Sensor Capacitance Estimates

According to the parallel plate capacitance model, the capacitance between two parallel plates is proportional to the overlapping area between the electrodes ($A=T_{e}\cdot w_{e}$) and inversely proportional to the electrodes gap ($D_{e}$) as follows,

(11) $$\begin{array}{c} C=\varepsilon_{r}\varepsilon_{0}\frac{T_{e}\;\cdot \;w_{e}}{D_{e}}, \end{array}$$

where $\varepsilon_{0}$ and $\varepsilon_{r}$ are the permittivity of the free space and the relative permittivity of the dielectric material between the two plates, respectively. As long as the electrode thickness ($T_{e}$) and width ($w_{e}$) are much bigger than the electrodes spacing ($D_{e}$), the electric field between the electrodes can be assumed uniform and the fringe fields are negligible. As either the electrode thickness or width becomes comparable to the electrode gap, the fringe fields may play an equally important role in the measured capacitance and the parallel plate estimates may thus deviates appreciably from the actual capacitance between the electrodes. Moreover, the parallel plate capacitance model given in Equation (11) does not account for the saturation of dielectric layer under increased compressive forces. Therefore, consistent with [31], and based on the experimental results as well as the FE simulations the correction factor in the form of $q{\left(U/D_{e}\right)}^{\gamma }$ is introduced in the change in capacitance expression to account for the above effects. As will be shown later on, *q* and γ are obtained through model comparison to the experimental results reported in [48] and non-linear curve fitting [51].

In light of the above and consistent with the flowchart shown in Figure 4b, the change in capacitance due to the sensor top-surface deformation can be calculated in terms of the original electrode thickness ($T_{e}$), current electrode thickness ($t_{e}$), original electrode gap ($D_{e}$), current electrode gap ($d_{e}$), and electrode width ($w_{e}$) as follows,

(12) $$\begin{array}{c} \Delta C=C-C_{0}=q\varepsilon_{r}\varepsilon_{0}w_{e}\left(\frac{t_{e}}{d_{e}}-\frac{T_{e}}{D_{e}}\right){\left(\frac{U}{D_{e}}\right)}^{\gamma }. \end{array}$$

Using the mechanics model developed in [49,50], the current electrode thickness and spacing can be calculated as follows,

(13) $$t_{e}=T_{e}-\left(u_{2}\left(x_{2}=S_{b}+T_{e}\right)-u_{2}\left(x_{2}=S_{b}\right)\right)$$

(14) $$d_{e}=D_{e}-u_{1}\left(x_{1}=\frac{D_{p}}{2},x_{2}=S_{b}\right)+u_{1}\left(x_{1}=\frac{D_{p}}{2}+D_{e},x_{2}=S_{b}\right).$$

## 4. Identification of Constitutive Parameters From the Force-Deformation Curves

As already mentioned, the substrate of the fabricated tactile sensor in [48] is modeled using the two-term Mooney-Rivlin (M-R) hyperelastic material response. One of the challenges in using hyperelastic material model, is obtaining the correct material parameters. While in our previous work [31] the M-R material parameters were estimated using the elastic moduli and incompressibility condition of PDMS, in this study, based on the methodology described in [52,53,54] an inverse method, combining finite element modeling and numerical optimization, is used to obtain the M-R material parameters $C_{1}$ and $C_{2}$. More specifically, first, based on the tactile unit-sensor geometry fabricated in [48] and shown in Figure 5, the related FE model with an initial guess of the M-R material parameters $x^{0}={\left[C_{1},C_{2}\right]}^{T}$ is developed and solved. Then, the force-deformation curves obtained from the FE simulations are compared to the ones from the experimental results. This is followed by an optimization process, where the M-R parameters are iteratively updated until the error between the FE simulations and experimental results becomes less than some tolerance threshold.

### 4.1. Finite Element Modeling

The commercial FE solver Abaqus [55] is used to carry out the FE simulations. Due to the large deformation and high non-linearity considered in this study, Abaqus/Explicit is used to solve the related quasi-static problem [49].

Consistent with an incompressible hyperelastic material model used in Section 2, the two-term M-R constitutive response is used to model the soft elastomeric layer. In Abaqus/Explicit, in order for the solver to overcome the convergence issues associated with ν=0.5, slight compressibility is assumed. However, as long as the layer is not highly confined, the slight introduced compressibility does not effect the accuracy of the solution [55].

It is noteworthy to mention that the M-R hyperelastic material model in Abaqus is defined slightly different than the model used in [49,50] and summarized in Section 2. More specifically, the M-R material parameters in Abaqus are defined as $C_{10}$ and $C_{01}$ and they are related to $C_{1}$ and $C_{2}$ defined earlier by the following expressions,

(15a) $$\begin{array}{c} C_{1}\left(\mathrm{in}\;\mathrm{model}\right)=2C_{10}\left(\mathrm{in}\;\mathrm{Abaqus}\right), \end{array}$$

(15b) $$\begin{array}{c} C_{2}\left(\mathrm{in}\;\mathrm{model}\right)=-2C_{01}\left(\mathrm{in}\;\mathrm{Abaqus}\right). \end{array}$$

For quasi-static simulations in Abaqus/Explicit, an appropriate step time needs to be determined such that the state of static equilibrium is achieved where the inertia effects can be neglected [55]. Consistent with [50], by performing frequency analyses, the step time period is set to 4 s for the simulations.

**Figure 6:**
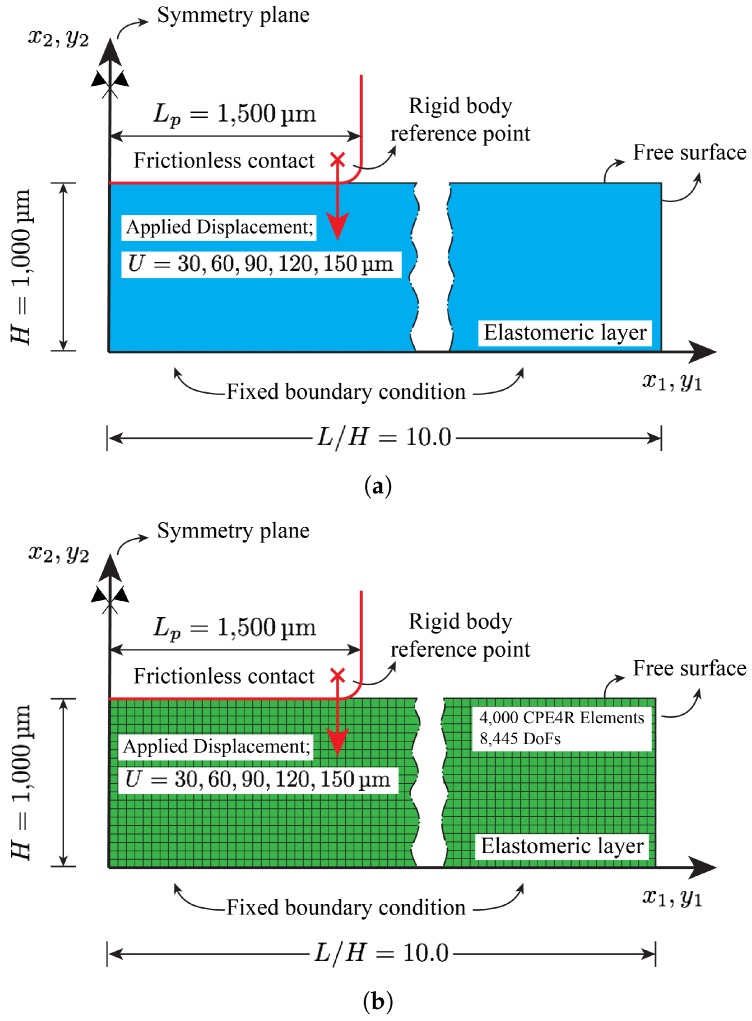
(**a**) The domain and associated boundary conditions used in carrying out the FE simulations; (**b**) The typical mesh used in FE analysis.

In accordance with the fabricated sensor geometry depicted in Figure 5, the discretized domain with associated boundary conditions shown in Figure 6 is used to perform the FE simulations. As shown in the figure, a 2-dimensional plane strain model is used. By employing symmetry boundary condition at $x_{1}=0$, only the right half of the polymeric layer is modeled. Initial layer thickness is set to *H* = 1000 μm. To ensure the infinite layer assumption, the aspect ratio of length to height L/H=10.0, resulting in total length of L=10.0mm, is chosen. Fixed boundary condition is used for the bottom surface of the polymeric layer at $x_{2}=0.0$. The punch is modeled as an analytic rigid body. The motion of a rigid body in Abaqus is governed by the motion of a single node, known as the rigid body reference node. Therefore, the prescribed displacements are applied to this node. More specifically, consistent with the experimental tests carried out in [48], increments of 30μm vertical displacements up to 150μm are applied to this node while keeping the horizontal displacement at zero. Also, as shown in Figure 6, to alleviate the convergence difficulties associated with the sharp corner of the rectangular punch, the punch’s corner is rounded accordingly.

The contact between the punch and the polymeric layer is modeled using a “contact pair” where the punch and the elastomeric layer are defined as a “master” and “slave” surfaces, respectively. Frictionless condition is assumed for the contact property so that the polymeric layer can freely slip below the punch.

The polymeric layer is discretize using a 4-node, bilinear, plane strain, quadrilateral and reduced elements known as CPE4R in Abaqus/Explicit. The typical mesh used in the FE simulations is shown in Figure 6b. Mesh convergence studies are carried out, such that further mesh refinement does not effect the solution considerably. The final mesh consists of 4000 elements corresponding to 4222 nodes and 8445 total degrees of freedom (1 Lagrange multiplier variable).

As will be shown later on, MATLAB optimization toolbox [51] is used in order to perform the non-linear optimization for finding the M-R material parameters. Therefore, a related Python script is written to carry out the FE simulations as needed to be compiled in the MATLAB environment. The script reads the M-R material parameters from a related file, performs simulations for all the displacements considered in this study (U=30,60,90,120,150μm), and reports the associated contact forces in the $x_{2}$-direction.

### 4.2. Inverse Analysis

As already mentioned MATLAB optimization [51] is used to solve a non-linear optimization problem. In doing so, Nelder-Mead simplex algorithm is used [56].

The optimization is formulated as the minimization of the objective function with respect to the unknown M-R material parameters $x={\left[C_{10},C_{01}\right]}^{T}$. The objective function Ψ(x) is defined as the square of the 2-norm of the error between the numerical results obtained by the FE simulations and experimental results reported in [48] i.e.,

(16) $$\begin{array}{c} \Psi \left(x\right)={e(x)}^{T}\;\cdot \;e(x)=\sum_{i=1}^{m}e_{i}^{2}\left(x\right), \end{array}$$

where m=5 is the number of the experimental data points (see Table 1) and $e_{i}$ is the error between the FE simulations and experimental measurements for the *i*th data point and is given as follows,

(17) $$\begin{array}{c} e_{i}\left(x\right)=F_{i}^{\mathrm{Exp}}-F_{i}^{\mathrm{FE}}\left(x\right). \end{array}$$

In the above expression, $F_{i}^{\mathrm{Exp}}$ and $F_{i}^{\mathrm{FE}}$ are the contact forces obtained for the *i*th displacement vector ($U={\left[30,60,\cdots ,150\right]}^{T}\;\mu m$) using experimental results and FE simulations, respectively.

It is noteworthy to mention that since the FE simulations are making use of the symmetry condition, in defining the objective function and calculating the error, either the experimental contact forces need to be divided by two to give the half of the contact forces corresponding to the symmetric model or the numerical results need to be multiplied by two to give the total contact force.

**Figure 7:**
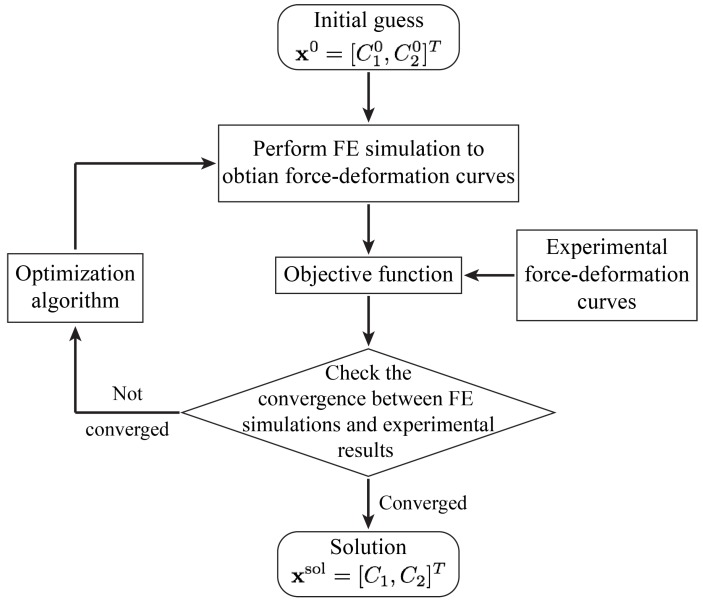
A flowchart of inverse FE optimization used in estimating the Mooney-Rivlin material parameters.

The inverse analysis starts with the initial guess of the M-R material constants, $x_{0}={\left[C_{10}^{0},C_{01}^{0}\right]}^{T}$. It then, performs the FE simulations using the above parameters, compares the results with experimental data points, and uses the optimization algorithm to iteratively update the solution until the FE and experimental results converges. The optimization procedure is summarized in the flowchart shown in Figure 7.

Using the inverse analysis with the initial material parameters $C_{10}$ = 4370 Pa and $C_{01}$ = 3670 Pa the optimization algorithm converges to the following solution after 12 iterations,

(18) $$\begin{array}{c} x_{\mathrm{sol}}={\left[C_{10},C_{01}\right]}^{T}={\left[70.25,39.53\right]}^{T}\;\mathrm{kPa}. \end{array}$$

**Figure 8:**
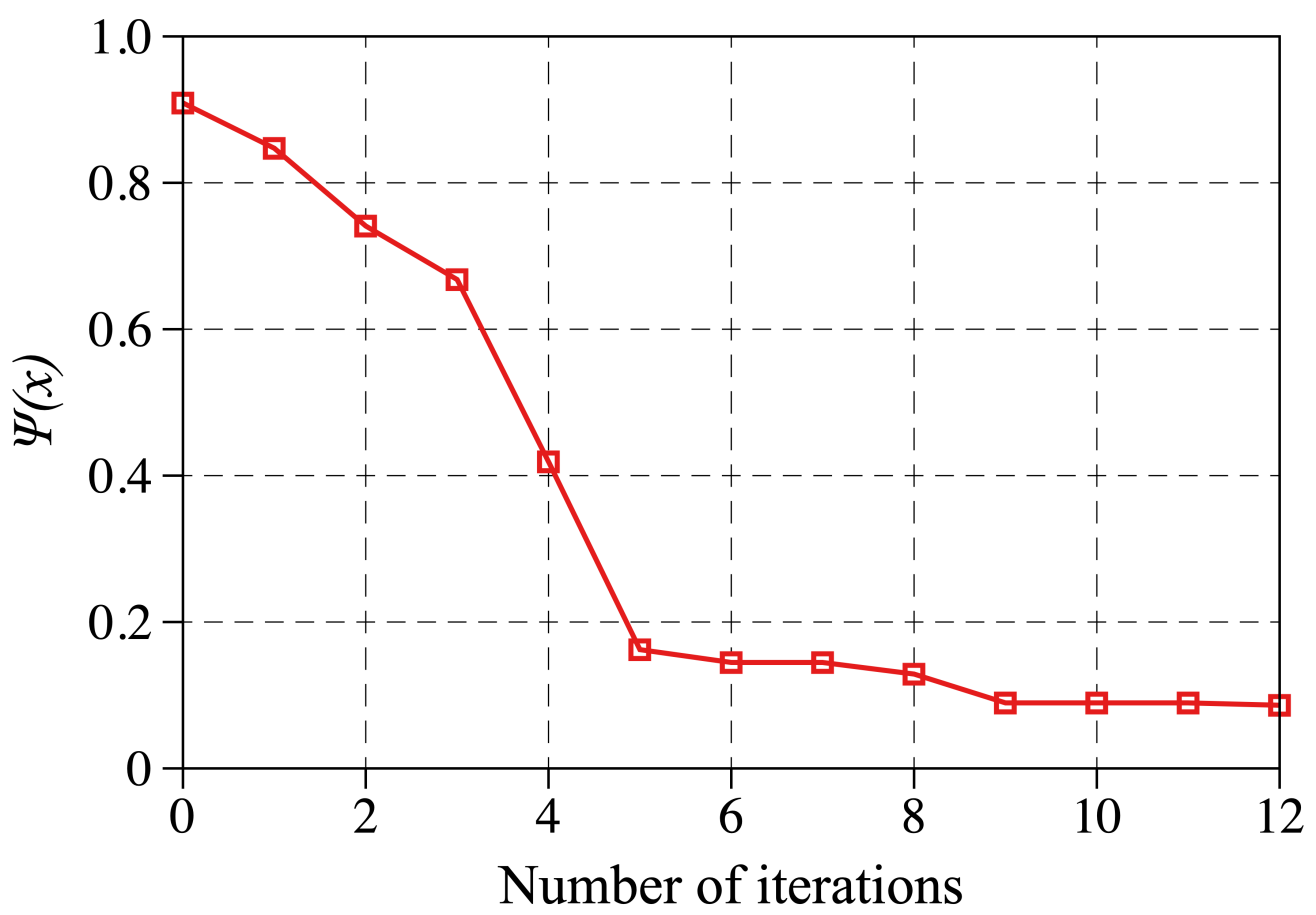
The objective function given by Equation (16) evaluated and plotted against the number of iterations to show the convergence behavior of the inverse problem employed in this study to find the M-R material parameters.

The objective function is evaluated at each iteration and is plotted against the number of iterations in Figure 8 to show the convergence behavior of the inverse problem. As shown, the error function drops very quickly during the first 8 iterations. It then hits a plateau and eventually stops after the 12th iteration.

**Figure 9:**
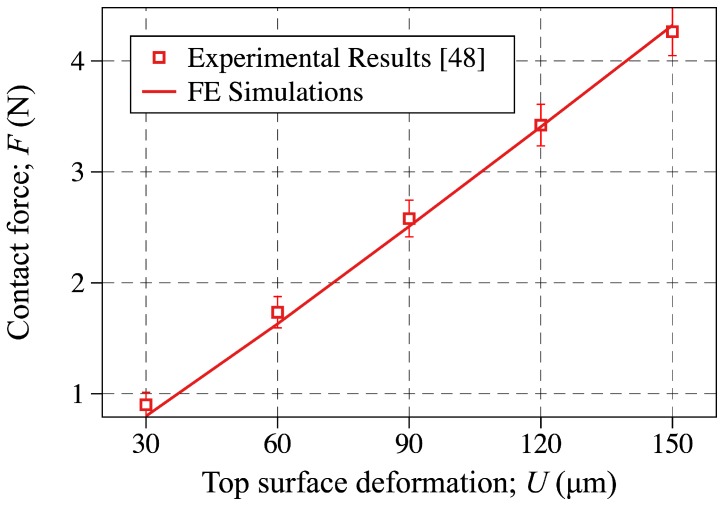
Probe contact force plotted against the top surface deformation obtained through experimental results reported in [48] (discrete points) and FE simulations carried out in this study (solid line). In obtaining the FE simulations the M-R material parameters obtained via the inverse analysis are used.

Using the parameters obtained by the inverse analysis, given in Equation (18), the contact force is plotted against the top-surface deformation and compared to the experimental results in Figure 9. More specifically, the discrete points are showing the experimental results reported in [48], while the solid line is showing the FE predictions using the M-R material parameters obtained through inverse analysis. As shown, the FE simulations are in great agreement with the experimental measurements, which indicates that the M-R material parameters given in Equation (18) captures the response of the PDMS layer used in the sensors fabricated in [48].

It is worth noting that the shear modulus corresponding to the above M-R material constants (Equation (18)) is calculated to be $\mu_{0}=2\left(C_{10}+C_{01}\right)=224.02\;\mathrm{kPa}$ which corresponds to elastic modulus of $E=3\mu_{0}=672.1\;\mathrm{kPa}$. The above is consistent with the shear and elastic modulus of PDMS reported in the literature [31,48].

In the following section, the tactile unit-sensor model presented in Section 3 will be used along with the M-R material parameters obtained through the inverse analysis to predict the response of these sensors. The model prediction will be then compared to the experimental results reported in [48] and summarized in Table 1.

## 5. Results and Discussion

The response of capacitive tactile sensors is captured through the change in capacitance-applied force curves. Accordingly, the ultimate goal of the modeling of these sensors is to establish the above relationship. Consistent with the flowchart shown in Figure 4, in this section, the aforementioned relationship will be formulated by combining the finite flat punch indentation model summarized in Section 2 with the capacitance model summarized in Section 3.3. More specifically, Equations (10) and (12) will be used to calculate the punch-sensor contact force and change in capacitance, respectively. As shown in the flowchart, for the change-in-capacitance calculations, the mechanics model given by Equation (1) is used to obtain the current electrode thickness and spacing. The parameters *q* and γ, accounting for fringe field and saturation effects, are obtained through model comparisons to the experimental results and non-linear curve fitting.

### 5.1. The Sensor-Probe Contact Force

**Figure 10:**
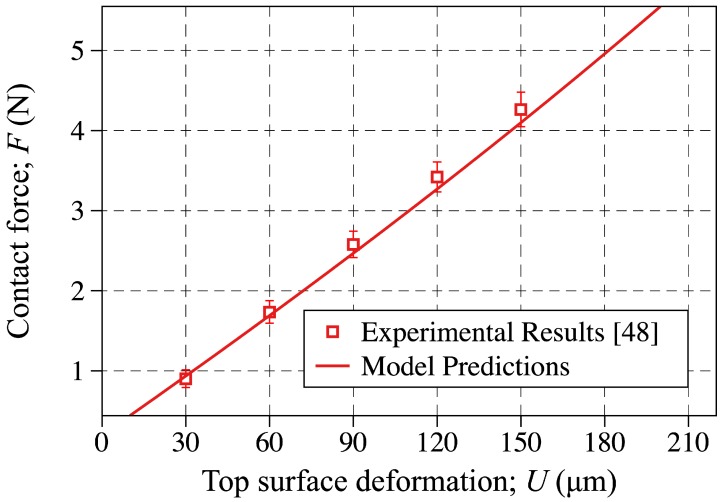
The probe contact force plotted against the top surface deformation level. The discrete points are showing the experimental result reported in [48], whereas the solid line is showing the modeling results obtained through Equation (10).

The reaction force developed in the contact probe due to the applied vertical displacement is plotted and compared to the experimental measurements reported in [48] in Figure 10. The discrete points are showing the experimental results (Table 1) and the solid line is showing the model prediction obtained by Equation (10). As shown, great agreements are found out to exist between the model predictions and the experimental results. This confirms that the finite flat punch indentation semi-analytical model developed in [50] and summarized in Section 2 can accurately predict the contact force applied on these all-elastomer sensors.

### 5.2. The Change in Capacitance-Applied Force Curves

**Figure 11:**
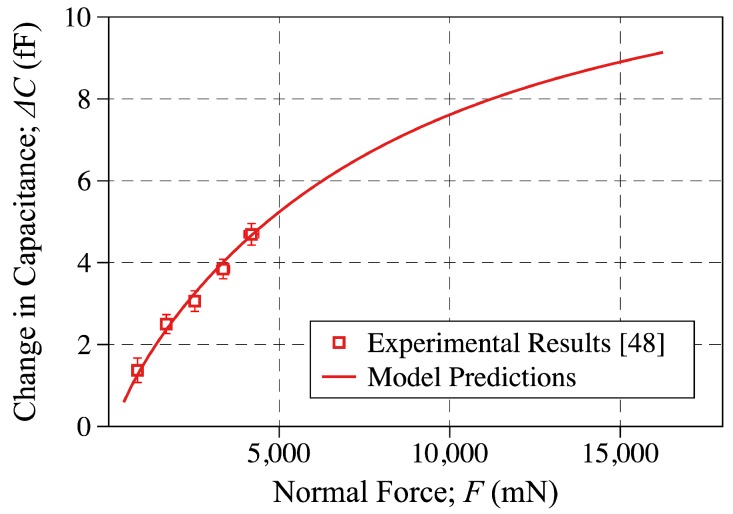
The change-in-capacitance plotted against the applied force. The discrete points are showing the experimental measurements reported in [48], while the solid line is showing the modeling results obtained in this study. The model predictions are reported for q=0.2469 and γ=−0.2720.

As already mentioned and consistent with [31], the $q{\left(U/D_{e}\right)}^{\gamma }$ term is introduced in the change-in-capacitance formula obtained by the parallel plate capacitance model to account for the fringe field and saturation effects. By using the experimental change-in-capacitance measurements and the non-linear curve fitting the above parameters were determined to be q=0.2469 and γ=−0.2720. Using the above parameters, the change-in-capacitance predicted by the model is plotted against the applied force and compared to the experimental results in Figure 11. In doing so, the applied force and change in capacitance are calculated using Equations (10) and (11), respectively. As shown, great agreement is found to exist between the model predictions and experimental measurements reported in [48]. It is worth noting that while the experimental results are reported for displacements of up to U=150μm [48], the model predictions shown in Figure 11 are obtained for top-surface deformation levels of up to U=500μm.

Consistent with the figure, as the applied force increases the capacitance change (slope of the F−ΔC curves) decreases. This agrees with our earlier findings in [31] and is due to the saturation of dielectric layer under increased applied forces as well as the deformation characterestics under large applied sensor displacement.

**Figure 12:**
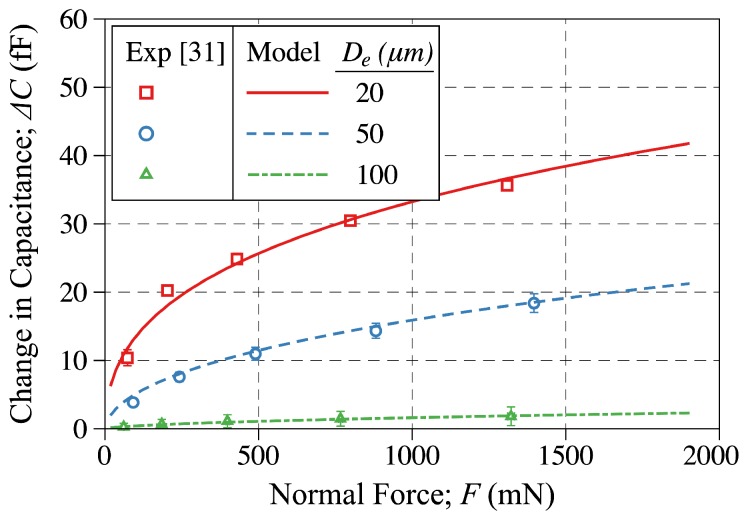
The change-in-capacitance plotted against the applied force. The discrete points are showing the experimental measurements reported in [31], while the solid line is showing the modeling results obtained in this study. The model predictions are reported for q=2.52, 2.10, 0.42 and γ=−0.27, −0.07, 0.13 for electrode gaps of $D_{e}=20,\;50,$ and 100 μm, respectively.

To further explore the model’s capabilities, the experimental capacitance change results obtained by testing three different sensors reported in [31] are plotted in Figure 12 along with the calibrated model predictions. As discussed in [31], three different sensors of electrode gap of 20, 50 and 100 μm were tested and the applied force, capacitance change were recorded. In generating the model predictions shown in Figure 12, the model was calibrated to match the layer geometry, electrode location and electrode gap along with the layer material properties as reported in [31]. As shown, the model predictions are in close agreement with the experimental results for all three sensors over their entire testing regime. This type of model comparison to experimental results reported both in Figure 11 and Figure 12 suggests that the model reported herein has the broad capabilities of capturing the tactile sensor response for a broad range of geometries, electrode gap and location, layer thickness and material properties. It is re-assuring to observe that while calibrating the model for one of the sensors reported in Figure 12, the model is then capable of capturing the response of the other two sensors by adjusting the electrode gap to match that of the corresponding sensor to which the model is compared to.

**Figure 13:**
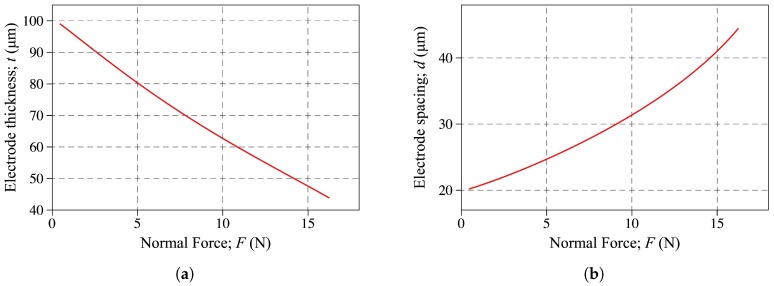
The electrode (**a**) thickness and (**b**) spacing plotted against the applied force. The results are obtained using the model developed in this study i.e., Equations (13) and (14).

To further understand the physics of the deformation, the electrode thickness and spacing is plotted against the applied force in Figure 13a,b, respectively. As expected, the electrode thickness decreases and the electrode spacing increases during the deformation.

Since extensive parametric studies have been carried out for different sensor geometrical parameters in our previous work [31], no such studies are presented in this work.

## 6. Conclusions

In this work, the non-linear finite flat punch semi-analytical model developed in [50] was combined with an enhanced capacitance model to develop a tactile unit-sensor model capable of predicting the sensor response under the application of normal forces over the sensor’s entire working range. In doing so both material and geometrical non-linearity assumed. Inverse analysis, combining finite element modeling and non-linear optimization, was performed to find the Mooney-Rivlin material constants $C_{1}$ and $C_{2}$. Using the experimental results and non-linear curve fitting, the enhanced capacitance model based on the parallel plate capacitance theory was developed to account for the fringe field as well as saturation effects. The model developed herein was validated by experimental results. In doing so, first, the applied force was plotted against the applied displacement and compared to the experimental results. Next, the change-in-capacitance was plotted against the applied force and also compared to the experimental measurements. Great agreements were found to exist between model predictions and experimental results in both curves.

Given the outcomes of this work, on-going studies are focused on the development of similar sensor models that account for both normal and shear forces. In addition, parametric studies on different sensor parameters (e.g., pillar height, width, placement, ⋯) need to be performed as needed to optimize the sensor geometry to achieve highest sensitivity and dynamic range under combined loading. Finally, the model and methodology employed in this study may also benefit other fields such as the field of biomaterials wherein tissue properties may be needed.

## Figures and Tables

**Table 1:** Experimental results associated with the application of normal force reported in [48].

*U* (μm)	*F* (N)	ΔC (fF)
30	0.9021	1.3710
60	1.7351	2.5004
90	1.5792	3.0618
120	3.4213	3.8438
150	4.2637	4.6920
